# Supplementary material for: Discovery of the major 15–30 nt mammalian small RNAs, their biogenesis and function
Source: Nat Commun. 2023 Sep 18;14:5796. doi: 10.1038/s41467-023-41554-6 (PMC10507107; doi:10.1038/s41467-023-41554-6)
Supplement: Supplementary file 1 — Supplementary Information [file 41467_2023_41554_MOESM1_ESM.pdf]

Supplementary Information

Discovery of the major 15-30 nt mammalian small RNAs, their biogenesis and function

Hejin Lai[1,3], Ning Feng[1,3] & Qiwei Zhai[1,2*]

[1] CAS Key Laboratory of Nutrition, Metabolism and Food Safety, Shanghai Institute of Nutrition and Health, University of Chinese Academy of Sciences, Chinese Academy of Sciences, Shanghai, China

[2] School of Life Science and Technology, ShanghaiTech University, Shanghai, China

[3] These authors contributed equally: Hejin Lai, Ning Feng

* Correspondence: qwzhai@sibs.ac.cn

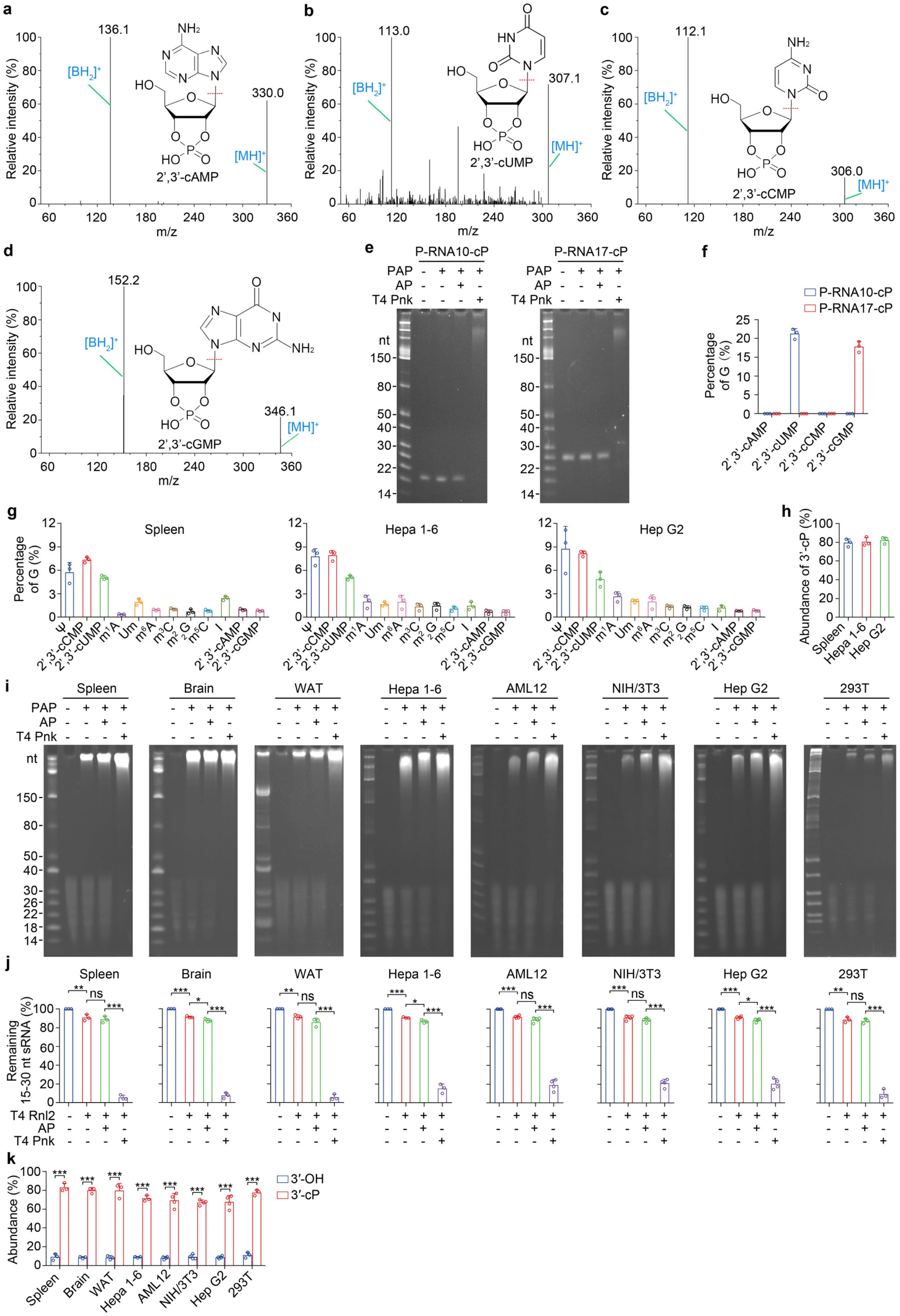

a
b
c
d
e
f
g
h
i
j
k
2',3'-cAMP
2',3'-cUMP
2',3'-cCMP
2',3'-cGMP
Relative intensity (%)
m/z
[BH2]+
[MH]+
136.1
330.0
113.0
307.1
112.1
306.0
152.2
346.1
P-RNA10-cP
P-RNA17-cP
PAP
AP
T4 Pnk
nt
Percentage of G (%)
Spleen
Hepa 1-6
Hep G2
Abundance of 3'-cP (%)
Brain
WAT
AML12
NIH/3T3
293T
Remaining 15-30 nt sRNA (%)
T4 Rnl2
ns
Abundance (%)
3'-OH
3'-cP

Supplementary Fig. 1. The 15-30 nt mouse and human sRNAs from various tissues and cell lines mainly end with 3'-cP.

(**a**-**d**) Electrospray ionization mass spectra of the indicated nucleotides. The quasimolecular ion peak $[MH]^+$ and/or product ion peak $[BH_2]^+$ of 2',3'-cAMP (**a**), 2',3'-cUMP (**b**), 2',3'-cCMP (**c**) and 2',3'-cGMP (**d**) are indicated.

(**e**) The synthetic sRNA-cPs including P-RNA-10-cP and P-RNA17-cP were analyzed by polyadenylation assay to confirm their 3'-cP modification.

(**f**) LC-MS/MS analysis of modified nucleotides from an enzymatic digestion of the synthetic sRNA-cPs including P-RNA-10-cP ended with 2',3'-cUMP and P-RNA17-cP ended with 2',3'-cGMP. n = 3.

(**g**) LC-MS/MS analysis of modified nucleosides and nucleotides from an enzymatic digestion of 15-30 nt sRNAs from the indicated tissue and cells. n = 3.

(**h**) The abundance of 15-30 nt sRNAs with 3'-cP from the indicated tissue and cells when analyzed by LC-MS/MS. n = 3.

(**i**) The 15-30 nt sRNAs from the indicated tissues and cells mainly end with 3'-cP when analyzed by polyadenylation assay. WAT, white adipose tissue. n = 3.

(**j**) Quantification of the remaining 15-30 nt sRNAs after polyadenylation in **i**. n = 3-4.

(**k**) The abundance of 15-30 nt sRNAs with 3'-OH and 3'-cP in the indicated tissues and cells detected and quantified in **j**, **k**. n = 3-4.

Data are presented as mean ± SD. Statistical significance was determined by two-tailed Student's t-test. *, $P < 0.05$; **, $P < 0.01$; ***, $P < 0.001$. ns, not significant. Exact P values can be found in Source Data Supplementary Fig. 1. Source data are provided as a Source data file.

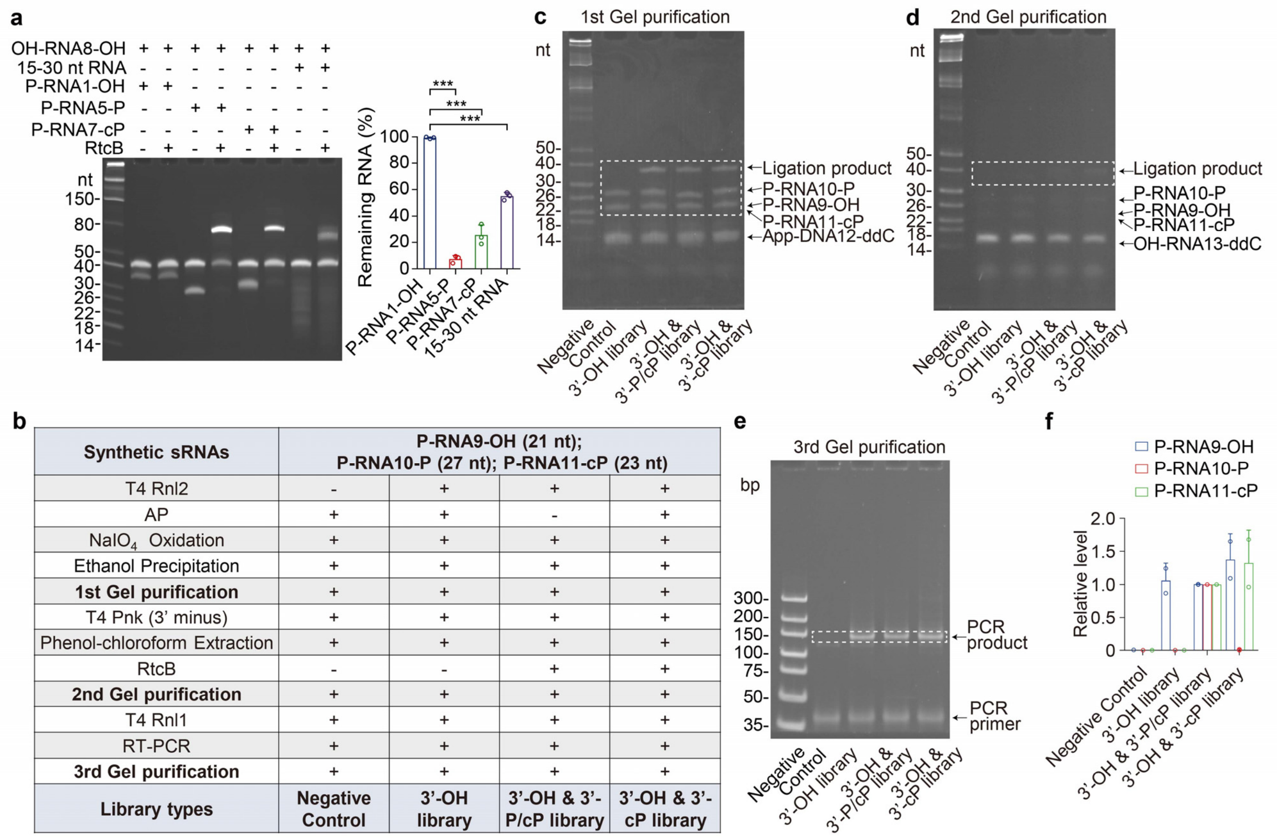

Synthetic sRNAs	P-RNA9-OH (21 nt); P-RNA10-P (27 nt); P-RNA11-cP (23 nt)			
T4 Rnl2	-	+	+	+
AP	+	+	-	+
$NaIO_4$ Oxidation	+	+	+	+
Ethanol Precipitation	+	+	+	+
**1st Gel purification**	+	+	+	+
T4 Pnk (3' minus)	+	+	+	+
Phenol-chloroform Extraction	+	+	+	+
RtcB	-	-	+	+
**2nd Gel purification**	+	+	+	+
T4 Rnl1	+	+	+	+
RT-PCR	+	+	+	+
**3rd Gel purification**	+	+	+	+
**Library types**	**Negative Control**	**3'-OH library**	**3'-OH & 3'-P/cP library**	**3'-OH & 3'-cP library**

Supplementary Fig. 2. Validation of the strategies for sRNA library construction.

(**a**) The synthetic sRNAs or 15-30 nt sRNAs from mouse liver with 3'-P or 3'-cP can be ligated by RtcB ligase. n = 3.

(**b**) Table summary of the key steps for the strategies of sRNA library construction.

(**c**) Denatured PAGE analysis after ligation with T4 Rnl2, dephosphorylation with or without AP, $NaIO_4$ oxidation and ethanol precipitation showed that the sRNAs with 3'-OH can be ligated with the 3'-adapter App-DNA12-ddC. The portion of gel within the rectangle was recovered.

(**d**) Denatured PAGE analysis after further phosphorylation with T4 Pnk (3' phosphatase minus), phenol-chloroform extraction and ligation with RtcB ligase showed that the sRNAs with 3'-P and 3'-cP can be ligated with the 3'-adapter OH-RNA13-ddC.

(**e**) Native PAGE analysis after further ligation with T4 RNA ligase 1 (T4 Rnl1) and RT-PCR showed that the indicated libraries can be successfully amplified.

(**f**) The constructed sRNA libraries were confirmed by qPCR to quantify the input synthetic RNAs. n = 2.

Data are presented as mean ± SD. Statistical significance was determined by two-tailed Student's t-test. *, $P < 0.05$; **, $P < 0.01$; ***, $P < 0.001$. Exact P values can be found in Source Data Supplementary Fig. 2. Source data are provided as a Source data file.

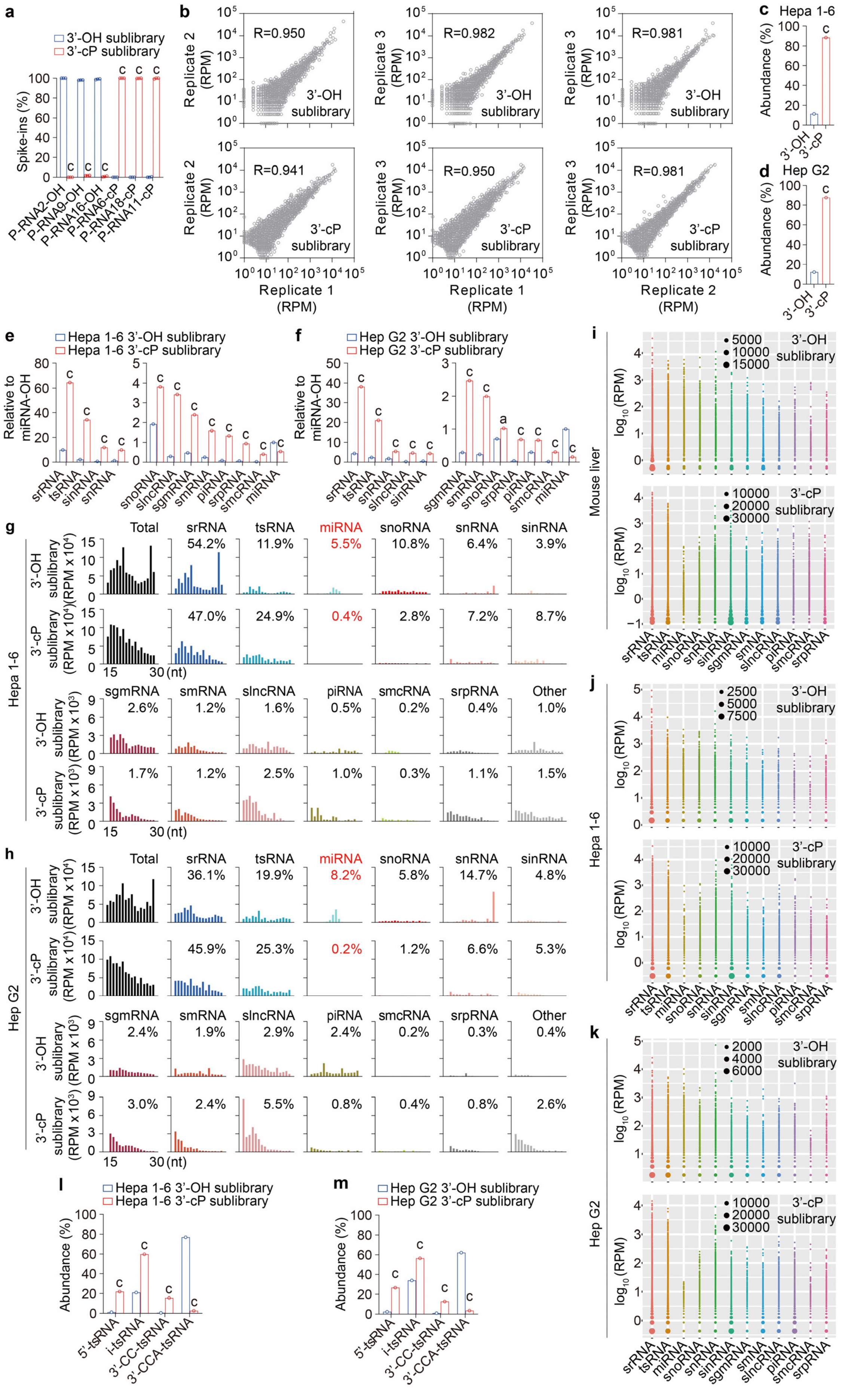

a
3'-OH sublibrary
3'-cP sublibrary
Spike-ins (%)
P-RNA2-OH
P-RNA9-OH
P-RNA16-OH
P-RNA6-cP
P-RNA18-cP
P-RNA11-cP
b
R=0.950
R=0.982
R=0.981
R=0.941
R=0.950
R=0.981
3'-OH sublibrary
3'-cP sublibrary
Replicate 1 (RPM)
Replicate 2 (RPM)
Replicate 3 (RPM)
c Hepa 1-6
d Hep G2
Abundance (%)
e
Hepa 1-6 3'-OH sublibrary
Hepa 1-6 3'-cP sublibrary
Relative to miRNA-OH
f
Hep G2 3'-OH sublibrary
Hep G2 3'-cP sublibrary
g
Hepa 1-6
Total srRNA tsRNA miRNA snoRNA snRNA sinRNA
54.2% 11.9% 5.5% 10.8% 6.4% 3.9%
47.0% 24.9% 0.4% 2.8% 7.2% 8.7%
sgmRNA smRNA slncRNA piRNA smcRNA srpRNA Other
2.6% 1.2% 1.6% 0.5% 0.2% 0.4% 1.0%
1.7% 1.2% 2.5% 1.0% 0.3% 1.1% 1.5%
h
Hep G2
36.1% 19.9% 8.2% 5.8% 14.7% 4.8%
45.9% 25.3% 0.2% 1.2% 6.6% 5.3%
2.4% 1.9% 2.9% 2.4% 0.2% 0.3% 0.4%
3.0% 2.4% 5.5% 0.8% 0.4% 0.8% 2.6%
3'-OH sublibrary (RPM x 10⁴)
3'-cP sublibrary (RPM x 10⁴)
3'-OH sublibrary (RPM x 10³)
3'-cP sublibrary (RPM x 10³)
15 30 (nt)
i Mouse liver
j Hepa 1-6
k Hep G2
log10 (RPM)
l
Hepa 1-6 3'-OH sublibrary
Hepa 1-6 3'-cP sublibrary
m
Hep G2 3'-OH sublibrary
Hep G2 3'-cP sublibrary
5'-tsRNA
i-tsRNA
3'-CC-tsRNA
3'-CCA-tsRNA

Supplementary Fig. 3. Cross-contamination, reproducibility and abundance analysis of 15-30 nt sRNAs with 3'-OH or 3'-cP.

(**a**) The relative abundance of the synthetic spike-ins shows no significant cross-contamination between 3'-OH and 3'-cP sublibraries. n = 3.

(**b**) Analysis of individual TANT-seq replicates for 3'-OH or 3'-cP sublibraries of 15-30 nt mouse liver sRNAs. Pearson correlation coefficient (R) indicated a high degree of overlap between individual replicates (R = 0.94 and above, as indicated on each plot).

(**c**-**d**) The relative abundance of 15-30 nt sRNAs with 3'-OH or 3'-cP from Hepa 1-6 (**c**) and Hep G2 cells (**d**).

(**e**-**f**) TANT-seq reveals many abundant sRNA biotype classes with 3'-OH or 3'-cP in Hepa 1-6 (**e**) and Hep G2 cells (**f**).

(**g**-**h**) Length distribution and abundance of 15-30 nt sRNAs in Hepa 1-6 (**g**) and Hep G2 cells (**h**).

(**i**-**k**) The distribution of abundance (y-axis) and unique reads (dot size) for sRNAs from the indicated sRNA biotype classes in 3'-OH and 3'-cP sublibraries of mouse liver (**i**), Hepa 1-6 (**j**) and Hep G2 cells (**k**).

(**l**-**m**) The percentage of tsRNA classified into each subgroup in 3'-OH and 3'-cP sublibraries of Hepa 1-6 (**l**) and Hep G2 cells (**m**) is significantly different.

Data are presented as mean ± SD. Statistical significance was determined by two-tailed Student's t-test or edgeR. a, $P < 0.05$; b, $P < 0.01$; c, $P < 0.001$. Exact *P* values can be found in Source Data Supplementary Fig. 3. Source data are provided as a Source data file.

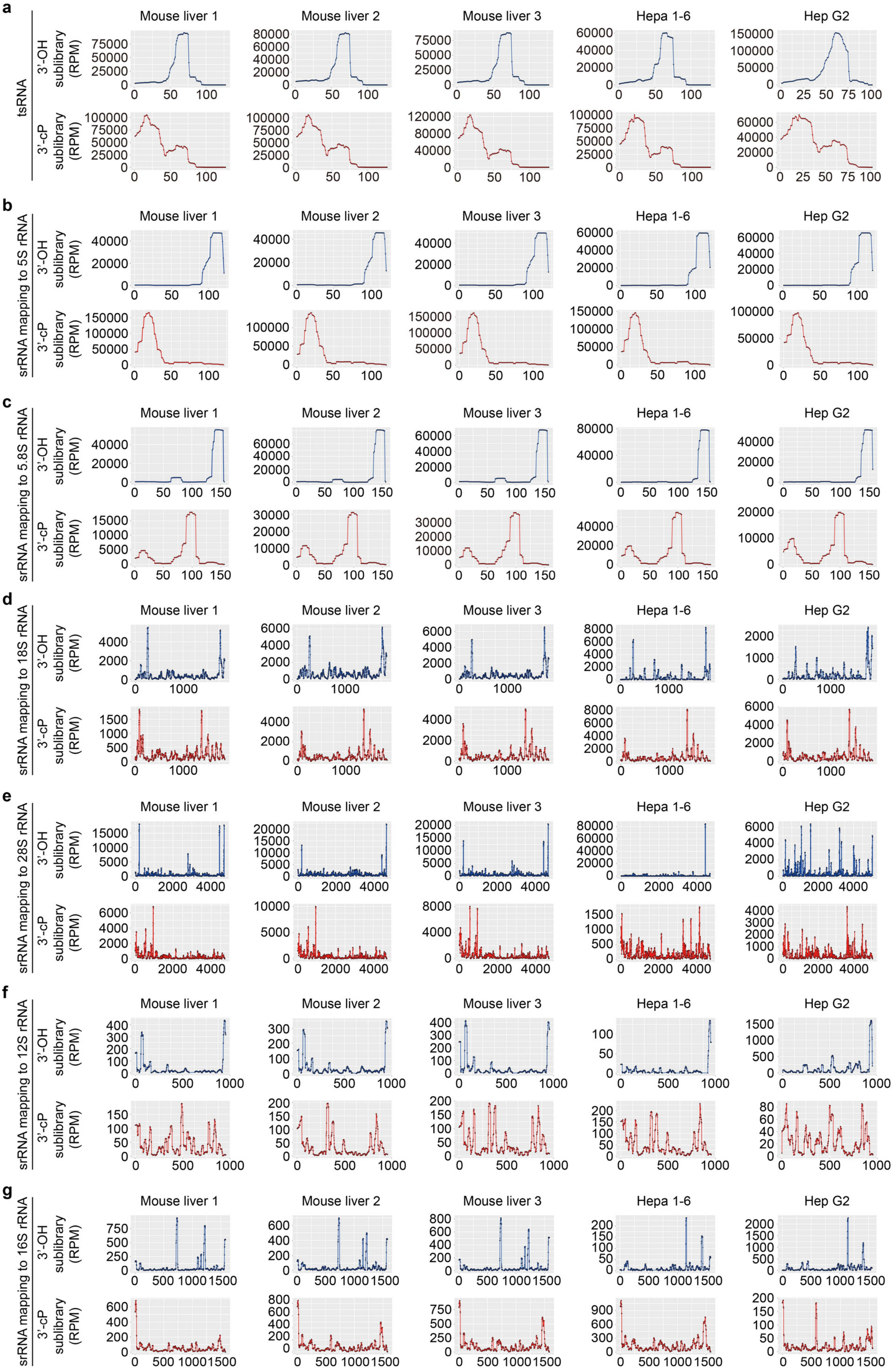

a
tsRNA
b
srRNA mapping to 5S rRNA
c
srRNA mapping to 5.8S rRNA
d
srRNA mapping to 18S rRNA
e
srRNA mapping to 28S rRNA
f
srRNA mapping to 12S rRNA
g
srRNA mapping to 16S rRNA
3'-OH sublibrary (RPM)
3'-cP sublibrary (RPM)
Mouse liver 1
Mouse liver 2
Mouse liver 3
Hepa 1-6
Hep G2

Supplementary Fig. 4. Overall length mapping of tsRNA and srRNA reads in 3'-OH and 3'-cP sublibraries.

(**a**) The distribution of tsRNA reads on a length scale in 3'-OH or 3'-cP sublibraries from mouse liver, Hepa 1-6 and Hep G2 cells.

(**b**-**g**) The distribution of srRNA reads mapped to 5S rRNA (**b**), 5.8S rRNA (**c**), 18S rRNA (**d**), 28S rRNA (**e**), 12S rRNA (**f**) and 16S rRNA (**g**) on a length scale in 3'-OH or 3'-cP sublibraries from mouse liver, Hepa 1-6 and Hep G2 cells.

Source data are provided as a Source data file.

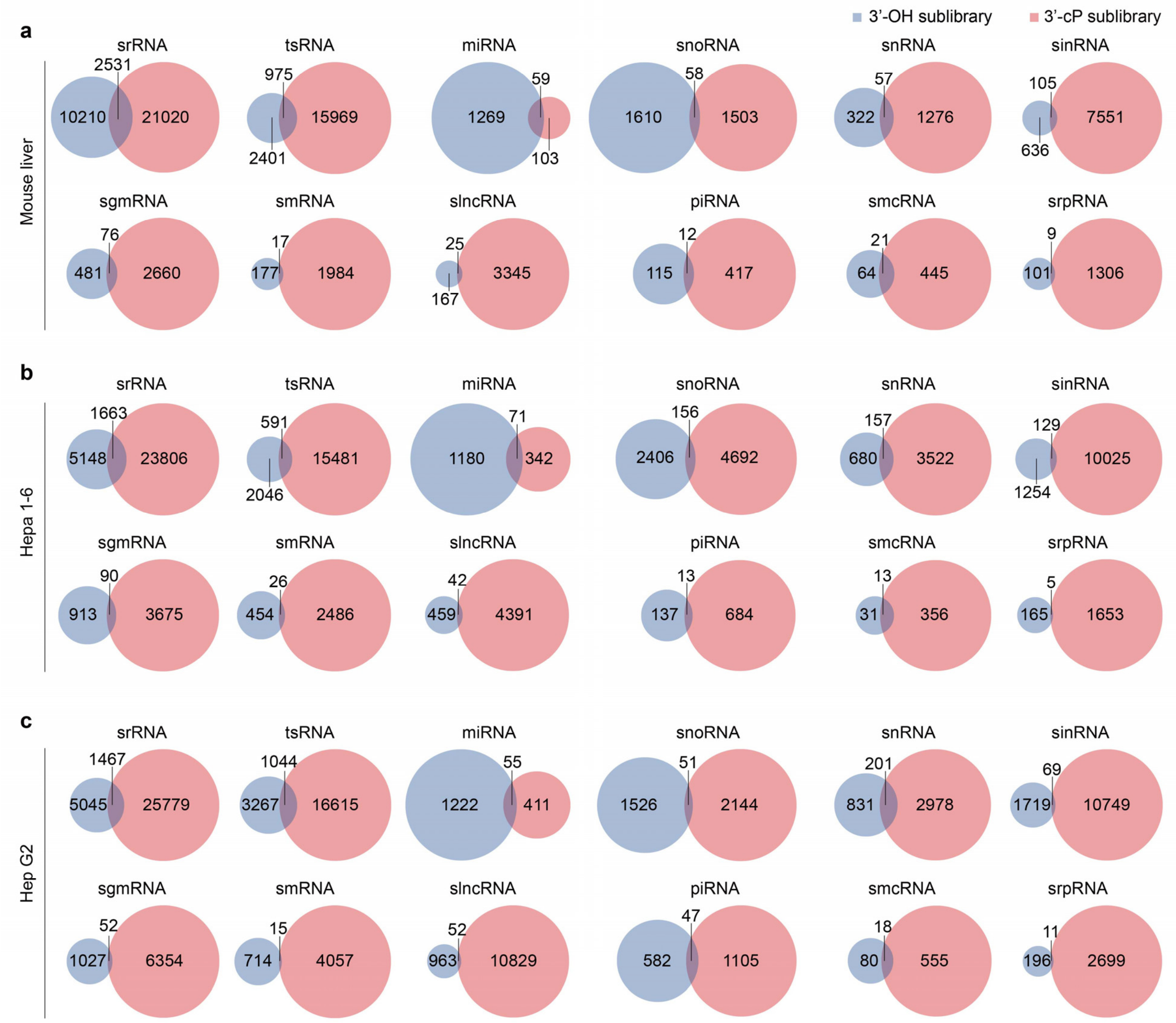

Supplementary Fig. 5. Overlap analysis of the indicated sRNA biotype classes in 3'-OH and 3'-cP sublibraries.

(**a**-**c**) The unique sRNAs between 3'-OH and 3'-cP sublibraries in the indicated biotype classes only have a little overlap in mouse liver (**a**), Hepa 1-6 (**b**) or Hep G2 (**c**) cells.

Source data are provided as a Source data file.

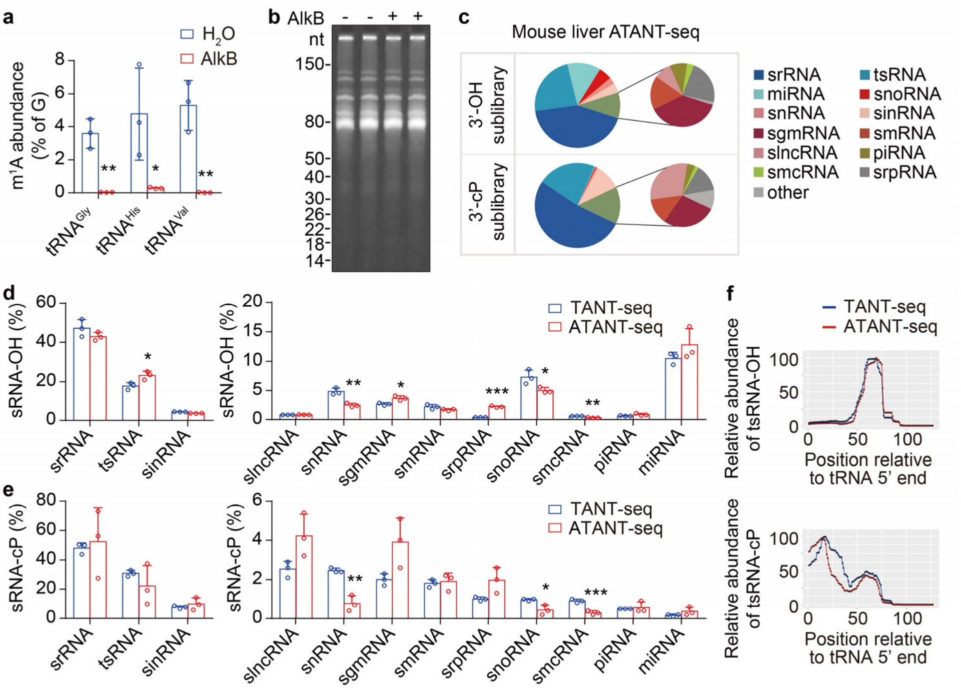

Supplementary Fig. 6. ATANT-seq reveals overall similar but slightly different sRNA expression profile compared to TANT-seq.

(**a**) AlkB treatment markedly reduced m^{1}A methylation in the indicated tRNAs. LC-MS/MS analysis of m^{1}A from an enzymatic digestion of the indicated tRNA purified from mouse liver treated with or without AlkB. n = 3.

(**b**) AlkB treatment didn't lead to significant degradation of sRNAs. Denatured PAGE analysis of sRNA (< 200 nt) from Hepa 1-6 cells treated with or without AlkB.

(c) Proportion of sRNA categories detected by ATANT-seq.

(**d-e**) ATANT-seq also reveals many abundant sRNA biotype classes with 3'-OH (**d**) or 3'-cP (**e**) as TANT-seq from mouse liver. n = 3.

(**f**) tsRNA-OH or tsRNA-cP reads obtained by TANT-seq and ATANT-seq from mouse liver were distributed on a length scale.

Data are presented as mean ± SD. Statistical significance was determined by two-tailed Student's t-test. *, P < 0.05; **, P < 0.01; ***, P < 0.001. Exact P values can be found in Source Data Supplementary Fig. 6. Source data are provided as a Source data file.

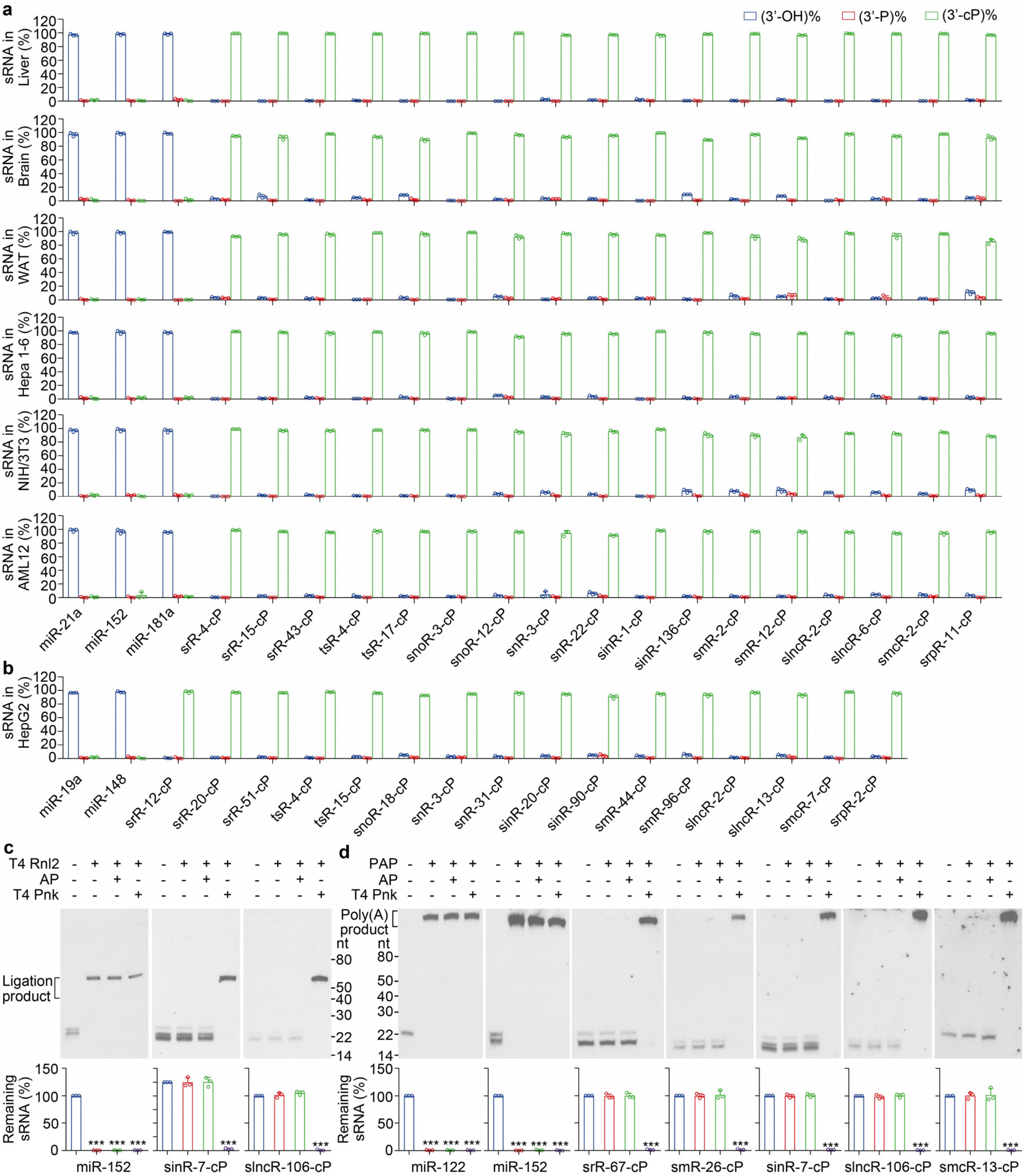

Supplementary Fig. 7. TE-qPCR and Northern blot to validate sRNAs detected by TANT-seq.

(**a**-**b**) The indicated sRNAs in mouse (**a**) and human (**b**) were detected by TE-qPCR. n = 3.

(**c**) The indicated sRNAs in mouse liver were detected by Northern blot after treatment with or without AP or T4 Pnk and ligation with or without T4 Rnl2, and the remaining sRNAs were quantified. n = 3.

(**d**) The indicated sRNAs in mouse liver were detected by Northern blot after treatment with or without AP or T4 Pnk and polyadenylation with or without Poly(A) polymerase (PAP), and the remaining sRNAs were quantified. n = 3

Data are presented as mean ± SD. Statistical significance was determined by two-tailed Student's t-test. *, $P < 0.05$; **, $P < 0.01$; ***, $P < 0.001$. Exact *P* values can be found in Source Data Supplementary Fig. 7. Source data are provided as a Source data file.

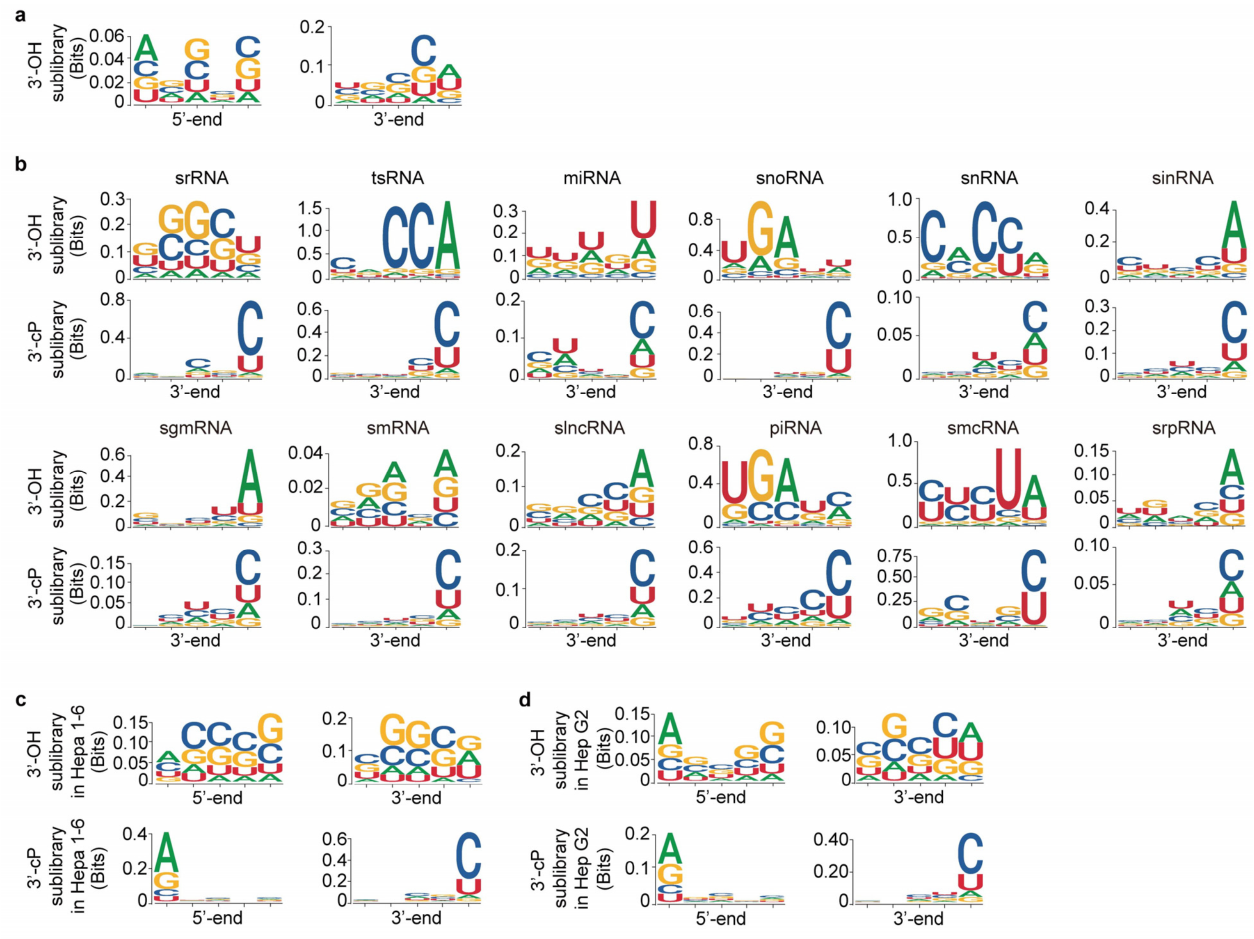

Supplementary Fig. 8. Nucleotide enrichment graphs for 15-30 nt sRNAs.

(**a**) Nucleotide enrichment graphs for 15-30 nt sRNAs with 3'-OH from mouse liver.

(**b**) Nucleotide enrichment graphs for the 3'-terminal of 15-30 nt sRNAs from mouse liver in the indicated categories.

(**c**) Nucleotide enrichment graph for 15-30 nt sRNAs from Hepa 1-6 cells.

(**d**) Nucleotide enrichment graph for 15-30 nt sRNAs from Hep G2 cells.

Source data are provided as a Source data file.

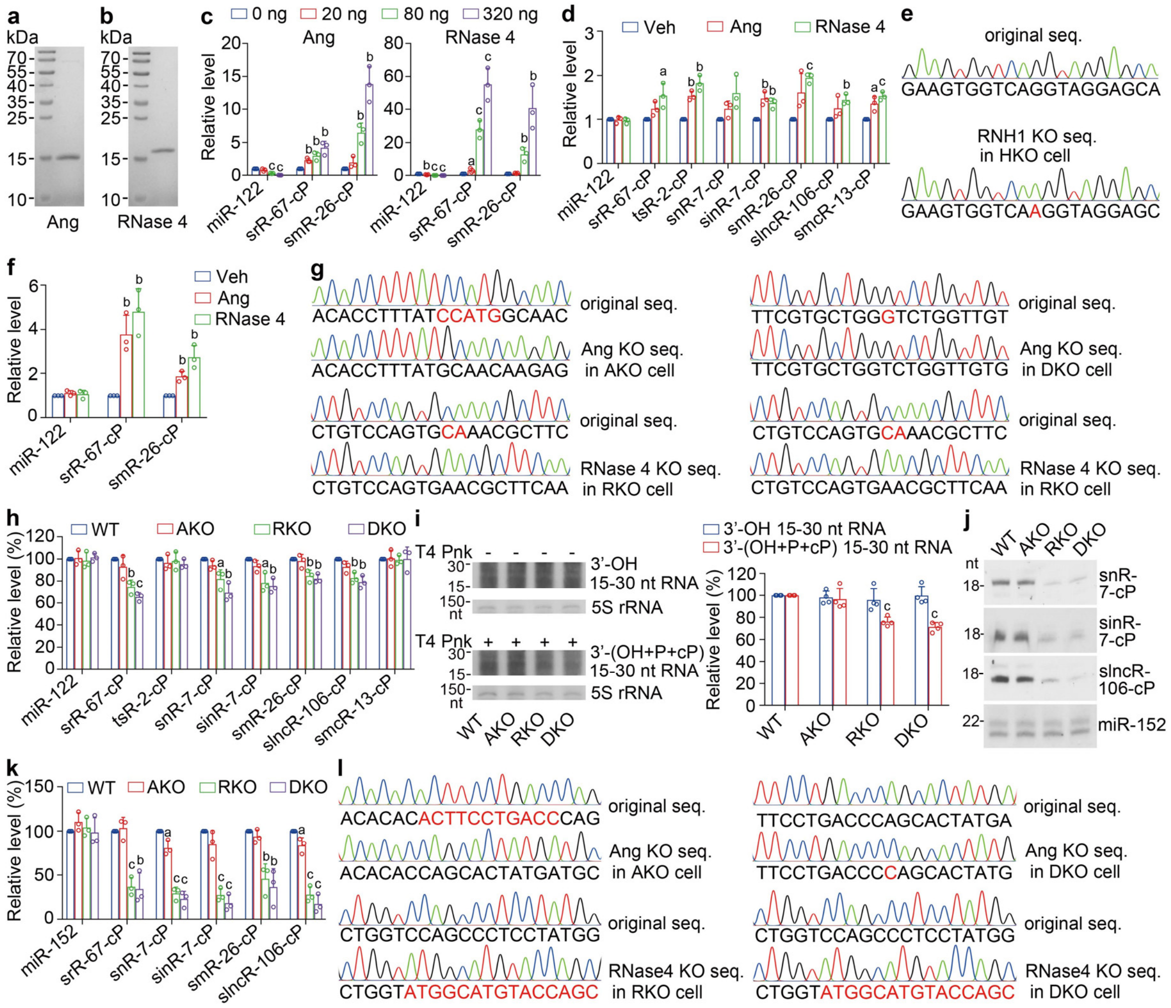

Supplementary Fig. 9. Ang and RNase 4 are responsible for the biogenesis of sRNA-cPs.

(**a**-**b**) The purified recombinant Ang (**a**) and RNase 4 (**b**) were confirmed by SDS-PAGE and Coomassie blue staining.

(**c**) Quantification of the sRNA levels after digestion with Ang or RNase 4 as shown in Fig. 4e. n = 3.

(**d**) Transfection with Ang or RNase 4 significantly increased the level of some sRNA-cPs in Hepa 1-6 cells when detected by TE-qPCR. n = 3.

(**e**) Chromatogram of DNA sequences in *RNH1* KO Hepa 1-6 cells, indicating successful genomic DNA mutation.

(**f**) Quantification of the sRNA levels after transfection with Ang or RNase 4 in *RNH1* KO Hepa 1-6 cells as shown in Fig. 4g. n = 3.

(**g**) Chromatogram of DNA sequences in *Ang* KO (AKO), *RNase 4* KO (RKO) and *Ang/RNase 4* double KO (DKO) Hepa 1-6 cells.

(**h**) The level of indicated sRNAs in WT, AKO, RKO and DKO Hepa 1-6 cells without pretreatment with RNase inhibitor when detected by TE-qPCR. n = 3.

(**i**) Detection and quantification of 15-30 nt sRNAs in WT, AKO, RKO and DKO Hepa 1-6 cells pretreated with RNase inhibitor. The level of 15-30 nt sRNAs was normalized to that of 5S rRNA. n = 4.

(**j**) The level of indicated sRNAs in WT, AKO, RKO and DKO Hepa 1-6 cells pretreated with RNase inhibitor when detected by Northern blot.

(**k**) Quantification of the indicated sRNAs detected by Northern blot in WT, AKO, RKO and DKO Hepa 1-6 cells. n = 3.

(**l**) Chromatogram of DNA sequences in AKO, RKO and DKO Hep G2 cells, indicating successful genomic DNA mutation.

Data are presented as mean ± SD. Statistical significance was determined by two-tailed Student's t-test. a, $P < 0.05$; b, $P < 0.01$; c, $P < 0.001$. Exact P values can be found in Source Data Supplementary Fig. 9. Source data are provided as a Source data file.

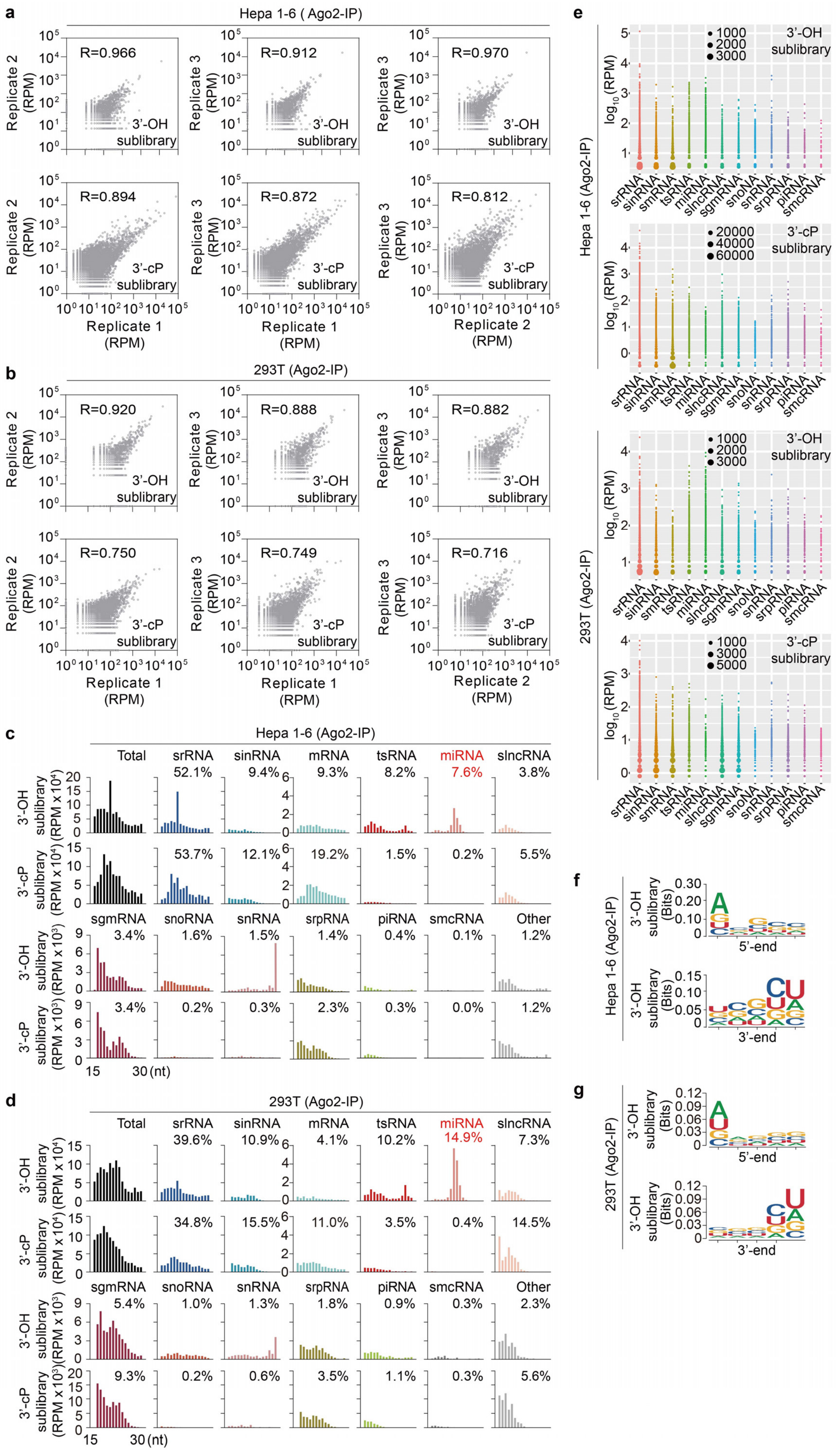
a
Hepa 1-6 (Ago2-IP)
R=0.966
R=0.912
R=0.970
R=0.894
R=0.872
R=0.812
3'-OH sublibrary
3'-cP sublibrary
Replicate 1 (RPM)
Replicate 2 (RPM)
Replicate 3 (RPM)
b
293T (Ago2-IP)
R=0.920
R=0.888
R=0.882
R=0.750
R=0.749
R=0.716
c
Hepa 1-6 (Ago2-IP)
Total
srRNA 52.1% 53.7%
sinRNA 9.4% 12.1%
mRNA 9.3% 19.2%
tsRNA 8.2% 1.5%
miRNA 7.6% 0.2%
slncRNA 3.8% 5.5%
sgmRNA 3.4% 3.4%
snoRNA 1.6% 0.2%
snRNA 1.5% 0.3%
srpRNA 1.4% 2.3%
piRNA 0.4% 0.3%
smcRNA 0.1% 0.0%
Other 1.2% 1.2%
3'-OH sublibrary (RPM x10⁴)
3'-cP sublibrary (RPM x10⁴)
3'-OH sublibrary (RPM x10³)
3'-cP sublibrary (RPM x10³)
15 30 (nt)
d
293T (Ago2-IP)
Total
srRNA 39.6% 34.8%
sinRNA 10.9% 15.5%
mRNA 4.1% 11.0%
tsRNA 10.2% 3.5%
miRNA 14.9% 0.4%
slncRNA 7.3% 14.5%
sgmRNA 5.4% 9.3%
snoRNA 1.0% 0.2%
snRNA 1.3% 0.6%
srpRNA 1.8% 3.5%
piRNA 0.9% 1.1%
smcRNA 0.3% 0.3%
Other 2.3% 5.6%
15 30 (nt)
e
Hepa 1-6 (Ago2-IP)
293T (Ago2-IP)
log10 (RPM)
1000 2000 3000
20000 40000 60000
1000 3000 5000
3'-OH sublibrary
3'-cP sublibrary
srRNA sinRNA smRNA tsRNA miRNA slncRNA sgmRNA snoNA snRNA srpRNA piRNA smcRNA
f
Hepa 1-6 (Ago2-IP)
3'-OH sublibrary (Bits)
5'-end
3'-end
g
293T (Ago2-IP)
5'-end
3'-end

Supplementary Fig. 10. Reproducibility, abundance and nucleotide enrichment analysis of 15-30 nt Ago2-binding sRNAs in Hepa 1-6 and 293T cells.

(**a**-**b**) Analysis of individual TANT-seq replicates for 3'-OH or 3'-cP sublibraries of 15-30 nt Ago2-binding sRNAs in Hepa 1-6 (**a**) and 293T cells (**b**).

(**c**-**d**) Length distribution and abundance of 15-30 nt Ago2-binding sRNAs in Hepa 1-6 (**c**) and 293T cells (**d**).

(**e**) The distribution of abundance (y-axis) and unique reads (dot size) for Ago2-binding sRNAs from the indicated sRNA biotype classes in 3'-OH and 3'-cP sublibraries of Hepa 1-6 and 293T cells.

(**f**-**g**) Nucleotide enrichment graph for 15-30 nt Ago2-binding sRNA-OHs in Hepa 1-6 (**f**) and 293T cells (**g**).

Source data are provided as a Source data file.

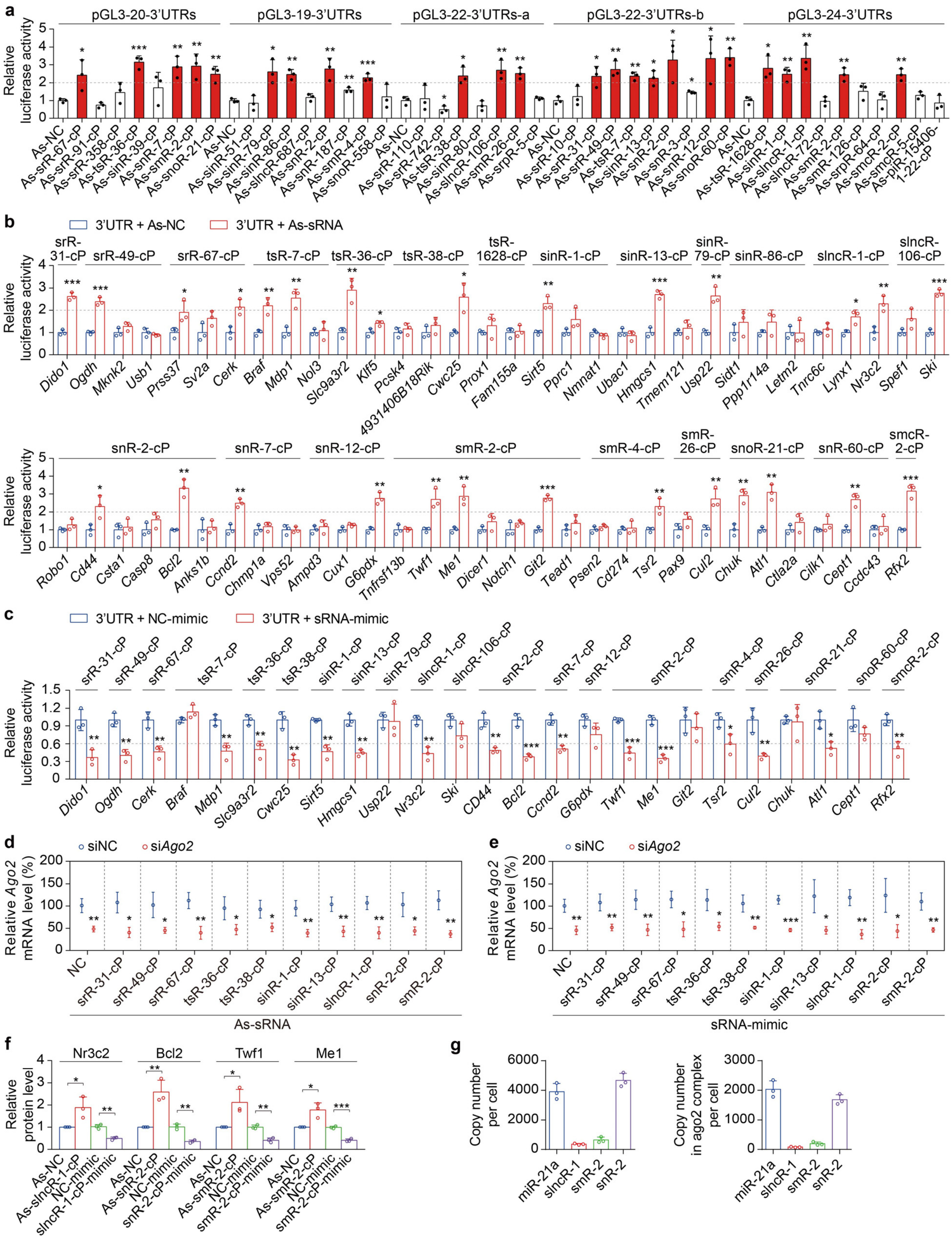

a
pGL3-20-3'UTRs
pGL3-19-3'UTRs
pGL3-22-3'UTRs-a
pGL3-22-3'UTRs-b
pGL3-24-3'UTRs
Relative luciferase activity
b
3'UTR + As-NC
3'UTR + As-sRNA
c
3'UTR + NC-mimic
3'UTR + sRNA-mimic
d
siNC
siAgo2
Relative Ago2 mRNA level (%)
As-sRNA
e
sRNA-mimic
f
Relative protein level
Nr3c2
Bcl2
Twf1
Me1
g
Copy number per cell
Copy number in ago2 complex per cell
miR-21a
slncR-1
smR-2
snR-2

Supplementary Fig. 11. Screen of functional sRNA-cPs regulating gene expression.

(**a**) Relative luciferase activity after transfection with the indicated luciferase plasmids containing the tandem predicted 3'UTRs and the indicated antisense sRNAs in Hepa 1-6 cells. n = 3.

(**b**) Relative luciferase activity after transfection with the luciferase plasmids containing 3'UTR of the indicated genes and the indicated antisense sRNAs in Hepa 1-6 cells. n = 3.

(**c**) Relative luciferase activity after transfection with the luciferase plasmids containing 3'UTR of the indicated genes and the indicated sRNA mimics in Hepa 1-6 cells. n = 3.

(**d**-**e**) The relative *Ago2* mRNA levels after transfection with siNC or si*Ago2* and the indicated antisense sRNAs (**d**) or sRNA mimics (**e**) in Hepa 1-6 cells. n = 3.

(**f**) Quantification of the indicated protein levels in Fig. 5e. n = 3.

(**g**) Quantification of miR-21a and the indicated sRNA-cPs in Hepa 1-6 cells or in Ago2 complex immunoprecipitated from Hepa 1-6 cells. n = 3.

Data are presented as mean ± SD. Statistical significance was determined by two-tailed Student's t-test. *, $P < 0.05$; **, $P < 0.01$; ***, $P < 0.001$. Exact P values can be found in Source Data Supplementary Fig. 11. Source data are provided as a Source data file.
