# Supplementary material for: Discovery of the major 15–30 nt mammalian small RNAs, their biogenesis and function
Source: Nat Commun. 2023 Sep 18;14:5796. doi: 10.1038/s41467-023-41554-6 (PMC10507107; doi:10.1038/s41467-023-41554-6)
Supplement: Supplementary file 3 — Description of Additional Supplementary Files [file 41467_2023_41554_MOESM3_ESM.pdf]

Description of Additional Supplementary Files

File Name: Supplementary Data 1
Description: Oligonucleotides used in this study.

File Name: Supplementary Data 2
Description: Calibration curves for LC-MS/MS analysis.

File Name: Supplementary Data 3
Description: 15-30 nt sRNA-OHs identified by TANT-seq in mouse liver or Hepa 1-6 cells.

File Name: Supplementary Data 4
Description: 15-30 nt sRNA-cPs identified by TANT-seq in mouse liver or Hepa 1-6 cells.

File Name: Supplementary Data 5
Description: 15-30 nt sRNA-OHs identified by TANT-seq in human Hep G2 cells.

File Name: Supplementary Data 6
Description: 15-30 nt sRNA-cPs identified by TANT-seq in human Hep G2 cells.

File Name: Supplementary Data 7
Description: Top 5000 15-30 nt Ago2-binding sRNA-OHs identified by TANT-seq in Hepa 1-6 cells.

File Name: Supplementary Data 8
Description: Top 5000 15-30 nt Ago2-binding sRNA-cPs identified by TANT-seq in Hepa 1-6 cells.

File Name: Supplementary Data 9
Description: Top 5000 15-30 nt Ago2-binding sRNA-OHs identified by TANT-seq in human 293T cells.

File Name: Supplementary Data 10
Description: Top 5000 15-30 nt Ago2-binding sRNA-cPs identified by TANT-seq in human 293T cells.

File Name: Supplementary Data 11
Description: 3’UTR luciferase reporter plasmids constructed in this study.
