# Supplementary material for: Discovery of the major 15–30 nt mammalian small RNAs, their biogenesis and function
Source: Nat Commun. 2023 Sep 18;14:5796. doi: 10.1038/s41467-023-41554-6 (PMC10507107; doi:10.1038/s41467-023-41554-6)
Supplement: Supplementary file 15 — Reporting Summary [file 41467_2023_41554_MOESM15_ESM.pdf]

nature research

Corresponding author(s): Qiwei Zhai

Last updated by author(s): Aug 31, 2023

Reporting Summary

Nature Research wishes to improve the reproducibility of the work that we publish. This form provides structure for consistency and transparency in reporting. For further information on Nature Research policies, see our Editorial Policies and the Editorial Policy Checklist.

Statistics

For all statistical analyses, confirm that the following items are present in the figure legend, table legend, main text, or Methods section.

n/a	Confirmed	
☐	☒	The exact sample size (*n*) for each experimental group/condition, given as a discrete number and unit of measurement
☐	☒	A statement on whether measurements were taken from distinct samples or whether the same sample was measured repeatedly
☐	☒	The statistical test(s) used AND whether they are one- or two-sided<br>*Only common tests should be described solely by name; describe more complex techniques in the Methods section.*
☒	☐	A description of all covariates tested
☒	☐	A description of any assumptions or corrections, such as tests of normality and adjustment for multiple comparisons
☐	☒	A full description of the statistical parameters including central tendency (e.g. means) or other basic estimates (e.g. regression coefficient) AND variation (e.g. standard deviation) or associated estimates of uncertainty (e.g. confidence intervals)
☐	☒	For null hypothesis testing, the test statistic (e.g. *F*, *t*, *r*) with confidence intervals, effect sizes, degrees of freedom and *P* value noted<br>*Give P values as exact values whenever suitable.*
☒	☐	For Bayesian analysis, information on the choice of priors and Markov chain Monte Carlo settings
☒	☐	For hierarchical and complex designs, identification of the appropriate level for tests and full reporting of outcomes
☐	☒	Estimates of effect sizes (e.g. Cohen's *d*, Pearson's *r*), indicating how they were calculated

Our web collection on statistics for biologists contains articles on many of the points above.

Software and code

Policy information about availability of computer code

Data collection: Analyst 1.6 software was used for LC-MS/MS data collection. Illumina Xten was used for sequencing data collection. CytExpert was used for flow cytometry data collection.

Data analysis: For high throughput sequencing data, raw reads were trimmed with fastp (version 0.20.0) , and the clean reads were sequentially mapped to the indicated reference database using ncbi-BLAST+2.11.0. R (version 4.0.5). For differential expression analysis of sRNAs, the fold changes and P values were calculated by Bioconductor package with edgeR. VennDiagram package was used to generate Venn diagrams. The sequence logos were constructed with the R package ggseqlogo. The distribution of abundance and unique reads for sRNAs was visualized in weighted scatter plots using the function geom_count from the package ggplot2 in R (version 3.3.5). Statistical analyses were performed with the GraphPad Prism 7.0 and Microsoft office Excel 2016.

For manuscripts utilizing custom algorithms or software that are central to the research but not yet described in published literature, software must be made available to editors and reviewers. We strongly encourage code deposition in a community repository (e.g. GitHub). See the Nature Research guidelines for submitting code & software for further information.

Data

Policy information about availability of data

All manuscripts must include a data availability statement. This statement should provide the following information, where applicable:

- Accession codes, unique identifiers, or web links for publicly available datasets
- A list of figures that have associated raw data
- A description of any restrictions on data availability

Raw sequence data have been deposited in the NCBI Sequence Read Archive under BioProject number PRJNA725316. Raw unprocessed LC-MS/MS data have been

deposited in Figshare (https://doi.org/10.6084/m9.figshare.23530554.v1). Source data are provided with this paper. All other data and materials are available upon reasonable request.

Field-specific reporting

Please select the one below that is the best fit for your research. If you are not sure, read the appropriate sections before making your selection.

☒ Life sciences ☐ Behavioural & social sciences ☐ Ecological, evolutionary & environmental sciences

For a reference copy of the document with all sections, see nature.com/documents/nr-reporting-summary-flat.pdf

Life sciences study design

All studies must disclose on these points even when the disclosure is negative.

Sample size	The sample size for each experiment is provided in the figure legends and in the main manuscript. Sample sizes were chosen to enable confident and meaningful conclusions from each experimental outcome. Except indicated, sample sizes of at least n = 3 biological replicates were used to determine significant differences in the changes observed in analyzed biological assays. No statistical methods were used to predetermine sample sizes. Sample size was based on empirical data from pilot experiments or previous reports. Examples: Hafner, M. et al., 2010 Cell; Shi, J. et al., 2021 Nat. Cell Biol..
Data exclusions	No data were excluded from the analysis.
Replication	All experiments were repeated (biological repeats) at least three times, except validation of the strategies for sRNA library construction was performed twice, and TANT-seq for cell samples was performed only once. All attempts at replication were successful.
Randomization	Experiments in replicates are under identical treatment conditions and sample preparation procedures. Randomization was not feasible for the in vitro studies. However, mice for fasting or high-fat diet experiment were randomly grouped.
Blinding	Investigators were not always able to be blinded due to limitations in available personnel.

Reporting for specific materials, systems and methods

We require information from authors about some types of materials, experimental systems and methods used in many studies. Here, indicate whether each material, system or method listed is relevant to your study. If you are not sure if a list item applies to your research, read the appropriate section before selecting a response.

Materials & experimental systems

n/a	Involved in the study	
☐	☒	Antibodies
☐	☒	Eukaryotic cell lines
☒	☐	Palaeontology and archaeology
☐	☒	Animals and other organisms
☒	☐	Human research participants
☒	☐	Clinical data
☒	☐	Dual use research of concern

Methods

n/a	Involved in the study	
☒	☐	ChIP-seq
☐	☒	Flow cytometry
☒	☐	MRI-based neuroimaging

Antibodies

Antibodies used	Anti-RNH1 (ABclonal, A4079, 1:5000)<br>anti-Angiogenin (Abcam, ab189207, 1:1000)<br>anti-RNase 4 (Abcam, ab200717, 1:1000)<br>anti-Tubulin (Sigma-Aldrich, T6074, 1:10000)<br>anti-AGO2 (Abcam, ab186733, 1:000)<br>anti-Bcl-2 (abclone, A19693, 1:1000)<br>anti-Nr3c2 (Abclonal, A3308, 1:1000)<br>anti-Twf1 (Abclonal, A15307, 1:1000)<br>anti-Me1 (Abclonal, A3956, 1:1000)<br>anti-Caspase-3 (Cell Signaling Technology, 9662S, 1:1000)<br>anti-Actin (Sigma-Aldrich, A1978, 1:10000)
Validation	Unless otherwise noted, all antibodies were validated by the manufacturer and used per their instructions. Additional information on validation can be found on the manufacturers' websites listed below:<br>Anti-RNH1 antibody (A4079) (https://abclonal.com.cn/catalog/A4079).<br>Anti-Angiogenin antibody (ab189207) (https://www.abcam.com/angiogenin-antibody-ab189207.html).<br>Anti-RNase 4 antibody (ab200717) (https://www.abcam.com/rnase4-antibody-ab200717.html).

Anti-Tubulin antibody (T6074) (https://www.sigmaaldrich.cn/CN/zh/search/t6074?focus=products&page=1&perPage=30&sort=relevance&term=T6074&type=product).
anti-AGO2 (ab186733) (https://www.abcam.com/argonaute-2-antibody-epr10411-ab186733.html)
anti-Bcl-2 (A19693) (https://abclonal.com.cn/catalog/A19693)
anti-Nr3c2 (A3308) (https://abclonal.com.cn/catalog/A3308)
anti-Twf1 (A15307) (https://abclonal.com.cn/catalog/A15307)
anti-Me1 (A3956) (https://abclonal.com.cn/catalog/A3956)
anti-Caspase-3 (9662S) (https://www.cellsignal.com/products/primary-antibodies/caspase-3-antibody/9662)
anti-Actin (A1978) (https://www.sigmaaldrich.cn/CN/zh/product/sigma/a1978)

Eukaryotic cell lines

Policy information about cell lines

Cell line source(s)	Mus musculus Hepa 1-6, AML12, NIH/3T3, Homo sapiens Hep G2 and 293T cells were obtained from National Collection of Authenticated Cell Cultures, CAS, China.
Authentication	Cell lines were not formally authenticated, but confirmation of expected gene expression patterns were performed by RNA-seq or qPCR.
Mycoplasma contamination	Cell lines were routinely checked for mycoplasma contamination.
Commonly misidentified lines (See ICLAC register)	No cell line used in this study was found in the database of commonly misidentified cell lines that is maintained by ICLAC.

Animals and other organisms

Policy information about studies involving animals; ARRIVE guidelines recommended for reporting animal research

Laboratory animals	To perform the fasting experiment, male C57BL/6 mice at the age of 8 weeks were randomly grouped and maintained in standard cages with or without access to food for 24 h, and water was available ad libitum in a room with an ambient temperature of 20-24°C and humidity of 40-60% on a 12-h light/dark cycle. To study the effect of high-fat diet, male C57BL/6 mice at the age of 8 weeks were randomly assigned to feed chow or high-fat diet with 60 kcal% fat (Research Diets) for 16 weeks. Male db/db mice at the age of 16 weeks were used.
Wild animals	No wild animals were involved in this study.
Field-collected samples	No field collected samples were involved in this study.
Ethics oversight	All animal studies were approved by the Institutional Animal Care and Use Committee of the Shanghai Institute of Nutrition and Health (ethics committee approval no. SIBS-2017-ZQW-1, SINH-2021-ZQW-1, SINH-2022-ZQW-1).

Note that full information on the approval of the study protocol must also be provided in the manuscript.

Flow Cytometry

Plots

Confirm that:

☒ The axis labels state the marker and fluorochrome used (e.g. CD4-FITC).

☒ The axis scales are clearly visible. Include numbers along axes only for bottom left plot of group (a 'group' is an analysis of identical markers).

☒ All plots are contour plots with outliers or pseudocolor plots.

☒ A numerical value for number of cells or percentage (with statistics) is provided.

Methodology

Sample preparation	Hepa 1-6 and HepG2 cells expressing mCherry were prepared to obtain single-cell clones. Hepa 1-6 cells were treated as indicated and stained with Annexin V-FITC and PI to analyze apoptotic cells.
Instrument	MoFlo Astrios EQ (Beckman Coulter) was used for cell sorting. CytoFLEX LX (Beckman Coulter) was used for analysis of Annexin V-FITC and/or PI positive cells.
Software	Summit v62 for cell sorting, and CytExpert for analysis of Annexin V-FITC and/or PI positive cells.
Cell population abundance	Abundance (Percentage) is indicated for the relevant populations on flow cytometry plots.

Gating strategy

For all experiments, FSC/SSC were used to discern single cells from doublets/multiple cells or cell debris. Cells without mCherry were used to establish boundaries between negative and positive cells for cell sorting. For analysis of Annexin V-FITC and/or PI positive cells, unstained, single stained and double stained cell controls were used to set gates and to eliminate negative cell population for each experiment.

☐ Tick this box to confirm that a figure exemplifying the gating strategy is provided in the Supplementary Information.
